# Intraspecific individual variation of temperature tolerance associated with oxygen demand in the European sea bass (*Dicentrarchus labrax*)

**DOI:** 10.1093/conphys/cov060

**Published:** 2016-01-08

**Authors:** Karlina Ozolina, Holly A Shiels, Hélène Ollivier, Guy Claireaux

**Affiliations:** af1 Core Technology Facility, The University of Manchester, 46 Grafton Street, Manchester M13 9NT, UK; af2 LEMAR, Unité PFOM-ARN, Centre Ifremer de Bretagne, Université de Bretagne Occidentale, Plouzané 29280, France

**Keywords:** Aerobic scope, climate change, fish, heart, individual variability, swimming

## Abstract

The European sea bass (*Dicentrarchus labrax*) is an economically important fish native to the Mediterranean and Northern Atlantic. Its complex life cycle involves many migrations through temperature gradients that affect the energetic demands of swimming. Previous studies have shown large intraspecific variation in swimming performance and temperature tolerance, which could include deleterious and advantageous traits under the evolutionary pressure of climate change. However, little is known of the underlying determinants of this individual variation. We investigated individual variation in temperature tolerance in 30 sea bass by exposing them to a warm temperature challenge test. The eight most temperature-tolerant and eight most temperature-sensitive fish were then studied further to determine maximal swimming speed (*U*_CAT_), aerobic scope and post-exercise oxygen consumption. Finally, ventricular contractility in each group was determined using isometric muscle preparations. The temperature-tolerant fish showed lower resting oxygen consumption rates, possessed larger hearts and initially recovered from exhaustive exercise faster than the temperature-sensitive fish. Thus, whole-animal temperature tolerance was associated with important performance traits. However, the temperature-tolerant fish also demonstrated poorer maximal swimming capacity (i.e. lower *U*_CAT_) than their temperature-sensitive counterparts, which may indicate a trade-off between temperature tolerance and swimming performance. Interestingly, the larger relative ventricular mass of the temperature-tolerant fish did not equate to greater ventricular contractility, suggesting that larger stroke volumes, rather than greater contractile strength, may be associated with thermal tolerance in this species.

## Teaser

Here we explored the hypothesis that individual variation in thermal tolerance in the European sea bass is associated with other key performance traits involved in determining individual Darwinian fitness. Our finding that whole animal temperature tolerance positively correlates with cardiac size could suggest selection on the cardiorespiratory system may benefit thermal tolerance.

## Introduction

The European sea bass (*Dicentrarchus labrax*) is an athletic predatory teleost fish found in the North Eastern Atlantic and the Mediterranean. Sea bass have an elaborate life cycle, which demands strong swimming performance and temperature tolerance. Adults spawn offshore in early spring, where the larvae hatch and then drift inshore (Pawson *et al.*, 2007). Juveniles form schools in sheltered estuaries, lagoons and other coastal areas, where they often experience broad daily and seasonal fluctuations in temperature, oxygen, salinity and turbulence (Pawson *et al.*, 2007). Once reproductively mature (after 4 years), adult sea bass inhabit highly dynamic environments, with high-energy tidal waves and currents, and undertake long annual migrations from inshore feeding grounds to offshore spawning grounds.

Swimming is energetically demanding, and the whole-body oxygen demand will change in response to changes in water temperature. Metabolic rate (MR) can be estimated by measuring the rate of oxygen consumption (${\dot{M}}_{O_{2}}$; see Clark *et al.*, 2013). Standard metabolic rate (SMR) is a measure of baseline aerobic metabolism required for life-sustaining processes, whereas the maximal metabolic rate (MMR) describes the maximal rate at which oxygen can be consumed by an organism. In athletic, fast-swimming fish species such as the sea bass, MMR is typically recorded during or immediately after strenuous exercise. The difference between MMR and SMR is the aerobic metabolic capacity or aerobic scope (AS) of the organism. Owing to their influence on SMR and MMR, environmental variables such as temperature and hypoxia (low O_2_) are liable to alter an organism's AS (Clark *et al.*, 2008; Eliason *et al.*, 2011; Anttila *et al.*, 2014) and, through prioritization of physiological functions, set the conditions for ecological performances (Fry, 1947). Therefore, AS has been proposed as a measure of whole-organism fitness in various environmental conditions (Soofiani and Priede, 1985; Claireaux and Lefrançois, 2007), including impending climate change (Pörtner and Knust, 2007; Pörtner and Farrell, 2008; Farrell *et al.*, 2009). However, in their natural environment, organisms are often affected by multiple stressors simultaneously, which can individually and collectively influence metabolism and thus their AS. Moreover, a reduction in AS can force fish to prioritize their energy demands depending on oxygen requirements and the fish's physiological status (i.e. nutritional state, sexual maturity, health etc.; Jourdan-Pineau *et al.*, 2010). However, the complexity and dynamics of such multidimensional environments are difficult to replicate and measure experimentally (Claireaux and Lefrançois, 2007).

There is a large degree of variation in MR and AS between individual fish within a population (e.g. Marras *et al.*, 2013; Killen *et al.*, 2014). Such variability also affects a broad range of physiological performances, including temperature tolerance (Anttila *et al.*, 2013) and swimming capacity (Reidy *et al.*, 2000; Yang *et al.*, 2015). In the sea bass, large inter-individual variation in swimming performance and thermal tolerance have also been reported and linked to survivorship in semi-natural field conditions (Nelson and Claireaux, 2005; Claireaux *et al.*, 2007, 2013; Handelsman *et al.*, 2010; Vandamm *et al.*, 2012). However, the factors underpinning intraspecific individual variation in thermal tolerance and swimming performance and their association with other important life-history traits have not been fully investigated. Nonetheless, a growing number of studies have implicated the oxygen supply and delivery abilities of the fish cardiovascular system as a key determinant of fish distribution and population resilience in a changing thermal environment (Pörtner and Farrell, 2008; Farrell *et al.*, 2009; Eliason *et al.*, 2011).

Fish possess a single-circuit cardiovascular system, where oxygen-depleted venous blood returning from the body enters the heart and is pumped to the gills for oxygenation. A consequence of this circulatory design is that the oxygen supply to the heart is provided by the oxygen remaining in venous blood after other tissues have extracted the oxygen they need. Moreover, many fish species, including the sea bass, do not possess a coronary blood supply (Icardo, 2012). Thus far, intraspecific variation in cardiac performance has been strongly linked to measures of whole-animal performance in a number of fish species. Claireaux *et al.* (2005) found that cardiac power output of rainbow trout (*Oncorhynchus mykiss*) with exceptional swimming performance was higher than that of poor swimmers, and Faust *et al.* (2004) reported intraspecific variation in cardiac tolerance of hypoxia in rainbow trout. Recently, Joyce *et al.* (2016) demonstrated that cardiac tolerance of hypoxia, assessed as isometric force production in isolated ventricular muscle preparations, was strongly correlated with whole-animal tolerance of hypoxia in sea bass.

The aim of this study was to investigate the physiological determinants of inter-individual variation in temperature tolerance and swimming performance indices in sea bass. We measured heat tolerance, maximal swimming speed (*U*_CAT_), SMR, MMR, post-exercise recovery time, excess post-exercise oxygen consumption (EPOC) and AS. We hypothesized that inter-individual variation in temperature tolerance would be correlated with oxygen demand characteristics and aerobic parameters. We also hypothesized that, through a common link with cardiac performance, there would be strong correlation in thermal tolerance and swimming performance in sea bass.

## Materials and methods

### Experimental animals

Thirty male European sea bass [average body mass 520 ± 64 g, range 419–643 g, body length (BL) 32 ± 1 cm, range 31–35 cm] were used for the study. The animals were obtained from a commercial fish farm (Aquastream, Lorient, France) and maintained at Institut Français de Recherche pour l′Exploitation de la Mer (IFREMER) in Brest, France. In order to differentiate between individuals, fish were anaesthetized (2-phenoxy-ethanol, 0.3 ml l^−1^) and tagged subcutaneously with a passive integrated transponder (PIT tag; Biolog-id, Paris, France). They were acclimated to the laboratory conditions for 9 months prior the start of the study in 2 m^3^ tanks (2 m × 2 m × 0.5 m) supplied with open-flow, aerated, bio-filtered sea water (salinity ∼32‰, temperature 10–13°C) in natural photoperiod conditions. Fish were fed daily on commercial dry pellets (Le Gouessant, Lamballe, France). Food was withheld for at least 24 h before each experiment. The experiments took place in November and December 2012, and all animal procedures were in accordance with the French and EU guidelines for animal research.

### Temperature challenge test

**Figure 1:**
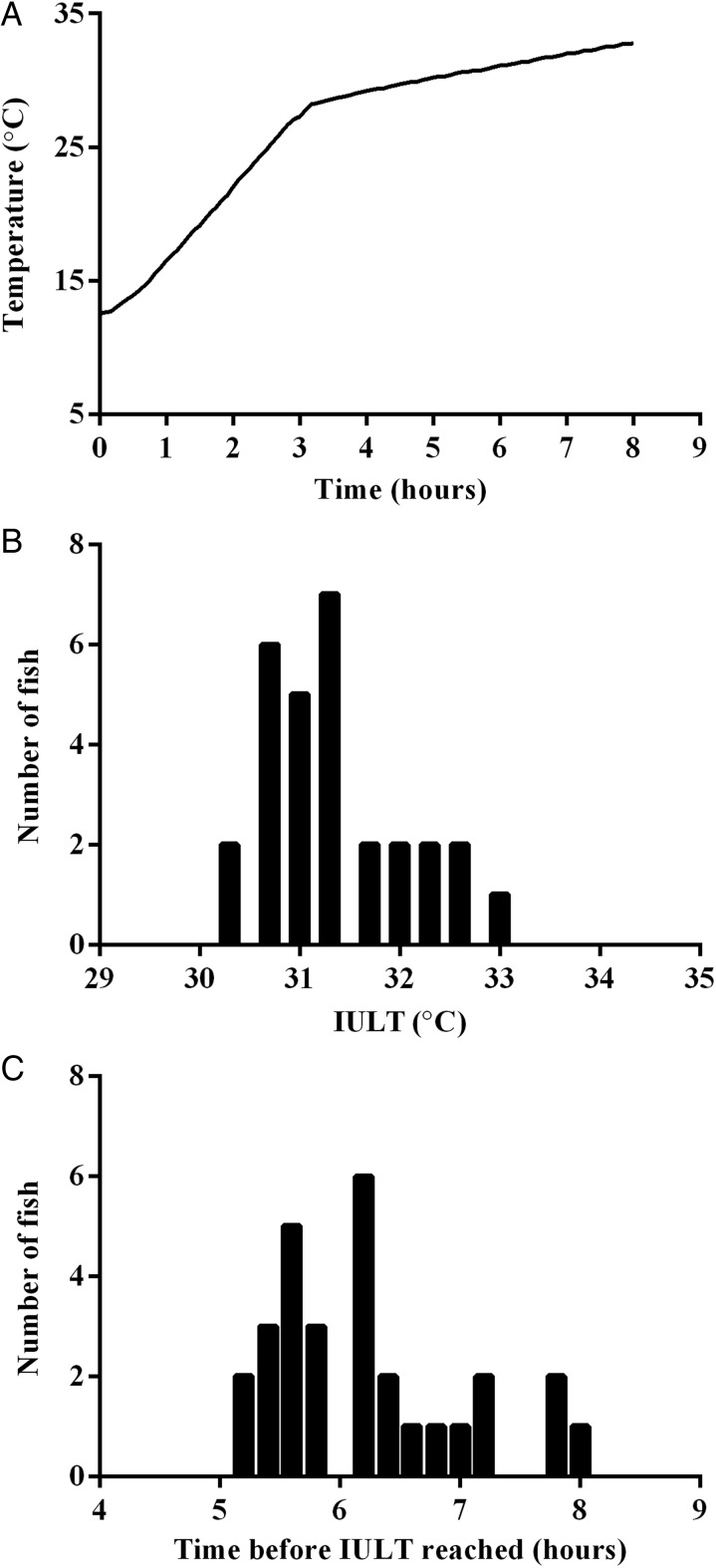
Individual variation in temperature tolerance in European sea bass. (**A**) Temperature tolerance was determined for 30 sea bass using a temperature challenge test. Initial warming was at a rate of 0.5°C min^−1^ from 13 to 27°C, followed by a slowed temperature change of 0.1°C min^−1^ from 27 to 32°C. For each fish, incipient upper lethal temperature (IULT) was recorded. These values were then binned into categories in the range of 0.3°C (**B**) and 0.2 h (**C**) to form population frequency histograms (*n* = 30).

Heat tolerance was assessed using a temperature challenge test (Claireaux *et al.*, 2013). Water in the fish housing tank was warmed up from 13 to 27°C at an approximate warming rate of 0.5°C min^−1^ using two 3 kW flow-through heaters (Van Gerven, Eindhoven, The Netherlands). At that point, the warming rate was decreased to 0.1°C min^−1^ (Fig. 1A). Throughout the temperature challenge test, water was bubbled with a mixture of air and oxygen gas to maintain water oxygenation at full saturation level. As the fish reached their maximal thermal limit (IULT), they lost their ability to maintain balance/equilibrium. As this occurred, they were rapidly removed from the experimental arena, scanned for identification and placed in a recovery tank at 13°C for observation (3 h) before being returned to their holding tank. All fish recovered fully from the temperature challenge test. The IULT and corresponding time were recorded for each individual, and the eight most temperature-tolerant and eight most temperature-sensitive individuals were selected for further studies.

### Constant acceleration swimming test

**Figure 2:**
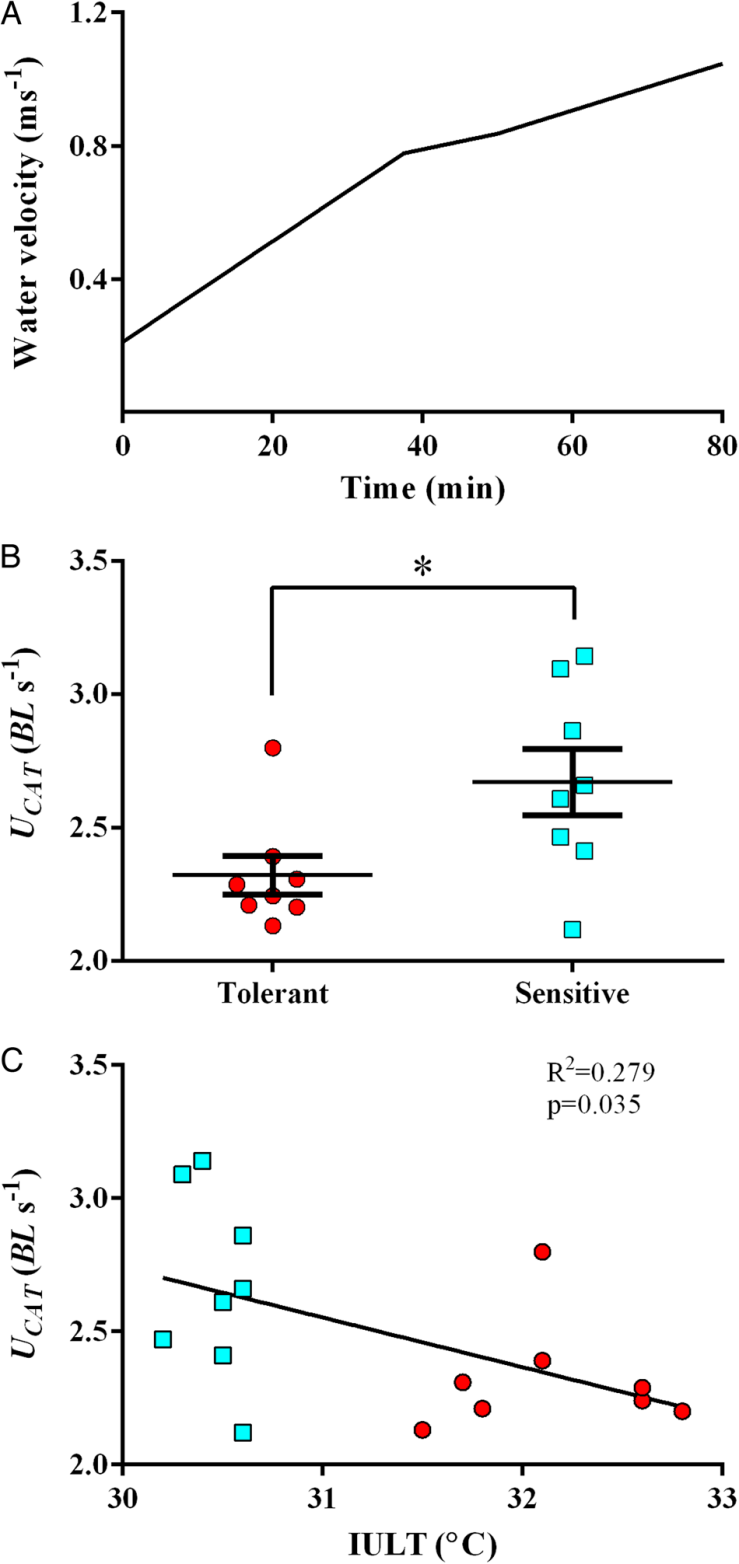
Temperature tolerance is negatively correlated with maximal swimming speed (*U*_CAT_). (**A**) In the constant acceleration test (CAT), water velocity was increased at an average rate of 0.15 m s^−1^ min^−1^ for 40 min, after which the rate was halved. (**B**) Mean *U*_CAT_ (i.e. water speed at which fish became exhausted) was significantly lower in the temperature-tolerant (red circles) group compared with the temperature-sensitive (blue squares) group (*t* = 2.42, *P* = 0.030); *n* = 8 for each group. (**C**) A negative correlation between individual temperature tolerance and swimming performance is shown for the most temperature-tolerant (red) fish and most temperature-sensitive fish (blue). Significance and *r*^2^-values were determined by Pearson's correlation coefficient (*n* = 16).

A constant acceleration test (CAT) was used to assess fish swimming capacity (Jain *et al.*, 1997; Reidy *et al.*, 2000; Marras *et al.*, 2010). From the subset of 16 fish (i.e. the eight temperature-tolerant and the eight temperature-sensitive individuals), four fish were chosen at random and placed in a cylindrical swimming flume (working area: 2 m length, 0.2 m diameter). Fish were acclimated to the flume for at least 3 h at a low water velocity [approximately 0.2–0.4 m s^−1^, the equivalent of ∼1 BL s^−1^] before the start of the CAT. The protocol was repeated until all 16 fish had undergone the CAT. Water flow was produced by a variable-speed pump (80/16-DE; Calpeda, Vicenza, Italy) connected to a frequency regulator (Mitsubishi, F700). Sea water was supplied to the swimming chamber from a tank containing thermoregulated and aerated water. The water velocity in the flume was measured using a hand-held flow probe secured in the flume (Hontzsch, Waiblingen, Germany). At the start of the CAT, water velocity was increased at a rate of 0.15 m s^−1^ min^−1^ for 40 min, after which the rate was halved (Fig. 2A). The fish shuffled position in the flume and did not interact physically with each other during the CAT. The anterior of the flume was enclosed in dark material to encourage upstream swimming. The animals swam until exhaustion, defined as the time point at which they would no longer maintain their position in the flume, did not respond to touch stimuli used to incite continuous swimming, and rested against the grid placed at the back of the working area. Exhausted fish were removed from the flume, identified via PIT tag and placed in a recovery tank for observation (3 h) before being returned to the holding tank. The water velocity at which the fish was exhausted was considered their maximal attainable swimming speed (*U*_CAT_). Water temperature in the flume was maintained at 12°C (±0.5°C). Swimming speeds were not corrected for solid blocking effects.

### Oxygen consumption

Oxygen consumption rates $\left({\dot{M}}_{O_{2}}\right)$ were measured using intermittent stop–flow respirometers. These respirometers were submerged in a larger container supplied with fully aerated and thermoregulated sea water (mean temperature 11 ± 0.5°C). For each trial, fish were removed from the rearing tank, placed in a 40 l container and stimulated to swim until exhaustion (∼5 min) by manual chasing prior to being introduced into one of the respirometry chambers (mean chamber volume 8.70 ± 0.13 l) for the next 48 h. The water oxygen saturation in the respirometers was monitored throughout and was always maintained above 80% air saturation. Water oxygenation was measured using an Oxy-4 oxygen meter (PreSens, Regensburg, Germany). Control of the flushing sequence and calculation of ${\dot{M}}_{O_{2}}$ were done automatically using AquaResp software (Aquaresp.com) and using the equation below (taken from Clark *et al.*, 2013):

$${\dot{M}}_{O_{2}}(\mathrm{mg}O_{2}kg^{-1}h^{-1})=\frac{\left[(V_{r}-V_{f})\times \Delta C_{WO_{2}}\right]}{\left(\Delta t\times M_{f}\right)},$$

where *V*_r_ is the respirometer volume, *V*_f_ is the fish volume (assuming that 1 g of fish is equivalent to 1 ml of water), $\Delta C_{wO_{2}}$ is change in oxygen concentration in the closed swim tunnel and *M*_f_ is fish mass in kilograms. The maximal metabolic rate was assumed to be the highest value of ${\dot{M}}_{O_{2}}$ and was typically one of the first data points from the respirometer. The SMR was calculated as the average of the 10% of the lowest ${\dot{M}}_{O_{2}}$ values for each animal. Aerobic scope was calculated as the difference between MMR and SMR. A first-order exponential equation was fitted to each individual's ${\dot{M}}_{O_{2}}$ curve to calculate EPOC (the area under the curve, in milligrams of O_2_ per kilogram), time to recovery (i.e. the time it took for each fish to return to RMR, in hours) and τ (the time constant of recovery, calculated as the time it took for the ${\dot{M}}_{O_{2}}$ to decrease by 63%, in hours; Weiss *et al.*, 1976; Nishimura *et al.*, 1993). At the end of the experiment, fish were returned to their holding tank and background oxygen consumption in empty respirometers was measured for 30 min and was found to be negligible. The respirometry set-up was cleaned between each experimental trial.

### Isometric myocardial preparations

The eight temperature-tolerant and eight temperature-sensitive individuals were killed with a sharp blow to the head followed by spinal cord severance. The heart was quickly excised and transferred to ice-cold isolation solution (pH 6.9) of the following composition (mM): NaCl, 100; KCl, 10; MgSO_4_, 4; KH_2_PO_4_, 1.2; taurine, 50; glucose, 20; and HEPES, 10. The ventricle was then isolated and weighed to calculate the relative ventricular mass (RVM, as a percentage, calculated as ventricle mass/animal mass × 100), before four ventricular muscle strip preparations were dissected free using a razor blade.

Four ventricular preparations from the same heart were run in parallel. Each muscle preparation was hung between two vertical clips; the uppermost was attached to a 25 g force transducer, while the other provided a stable anchor. Output from the force transducers was amplified (Transbridge 4M; World Precision Instruments, Sarasota, FL, USA) and converted to a continuously acquired digital signal using DataTrax data acquisition software (World Precision Instruments). The four muscle preparations were lowered into individual organ baths containing physiological solution (pH 7.7) of the following composition (mM): NaCl, 150; KCl, 5.4; MgSO_4_, 1.5; NaH_2_PO_4_, 0.4; CaCl_2_, 2.0; glucose, 10; and HEPES, 10. Half of the organ baths were maintained at 12°C and half at 27°C (chosen as a temperature close to the upper limit, but lower than any IULT). All organ baths were bubbled with oxygen throughout the experiment (Shiels and Farrell, 1997; Tiitu and Vornanen, 2002; Imbert-Auvray *et al.*, 2013).

The ventricular preparations were allowed to equilibrate to the organ bath for 20 min before stimulation (10 ms, 70 V, 0.2 Hz; Grass Student stimulator) commenced. Once the force of contraction had stabilized, the preparations were stretched to a length at which contraction was maximal (*L*_max_) and left at this length for a further 15 min before commencement of the stimulation frequency trial. Here, the stimulation frequency was increased from 0.2 Hz, briefly to 0.5 Hz and then to 0.8 Hz, where it was allowed to stabilize. We chose to look at 0.2 and 0.8 Hz because these frequencies bracket the *in vivo* range of sea bass heart rate at 12°C (Axelsson *et al.*, 2002).

### Statistical analysis

All statistical analyses were carried out in Prism (GraphPad Software, Inc., La Jolla, CA, USA). Incipient upper lethal temperature data were binned prior to displaying it in frequency histograms. Pearson's coefficient was calculated to measure the relationship between *U*_CAT_ and IULT. Student's unpaired *t*-tests were used to assess statistical differences in *U*_CAT_, SMR, MMR, AS, RVM and τ between the temperature-tolerant and temperature-sensitive groups. A two-way ANOVA was used to compare change in force of contraction in the ventricular preparations from the temperature-sensitive and temperature-tolerant fish at both experimental temperatures. All data are shown as scatter dot plots with individual values, means ± SEM unless stated otherwise. Significance was assumed at *P* < 0.05.

## Results

### Temperature tolerance

Sea bass showed individual variation in temperature tolerance, with IULT ranging from 30.2 to 32.8°C. This corresponded to nearly 3 h between loss of equilibrium in the most temperature-sensitive and the most temperature-tolerant fish (Fig. 1B and C). There were no significant differences between the body mass and BL of our temperature-tolerant and temperature-sensitive individuals (see online supplementary material Fig. S1A and B).

### Swimming capacity

The maximal speed achieved in the CAT (*U*_CAT_) varied greatly within our subset experimental population of 16 sea bass; the difference between the fastest and the slowest swimmer was in excess of 1 BL s^−1^ (3.14 vs. 2.12 BL s^−1^, respectively). The temperature-tolerant group had significantly lower mean *U*_CAT_ compared with the temperature-sensitive group, 2.32 ± 0.07 and 2.67 ± 0.12 BL s^−1^, respectively (*t* = 2.42, *P* = 0.030; Fig. 2B). When individual values of IULT were correlated with individual values of *U*_CAT_, a significant negative relationship was found (Pearson's correlation, *r*^2^ = 0.279, *P* = 0.035; Fig. 2C). Inter-individual *U*_CAT_ performance showed no relationship with fish size, RVM, ${\dot{M}}_{O_{2}}$ or τ (see online supplementary material Fig. S1A–F).

### Oxygen consumption

**Figure 3:**
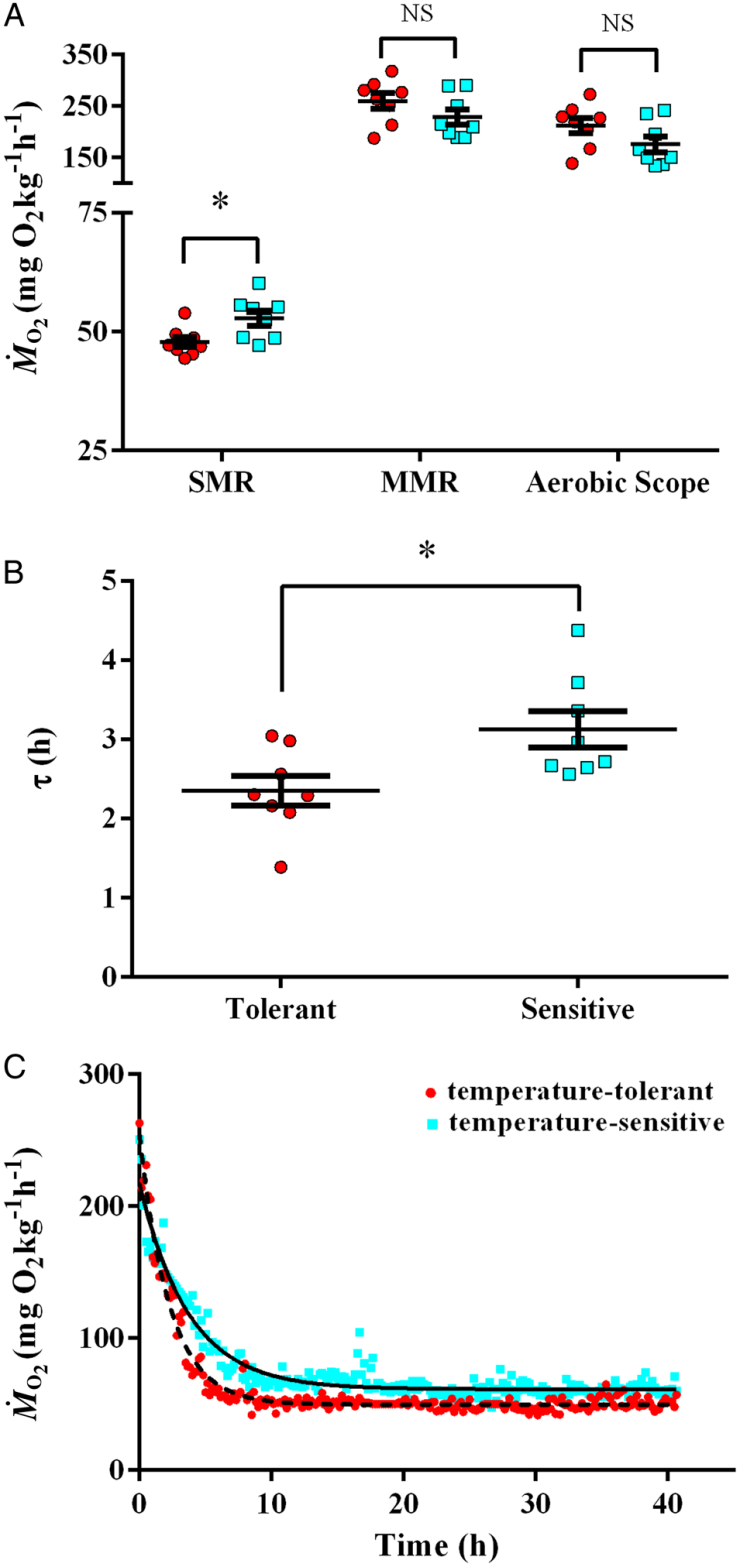
Temperature tolerance was associated with lower standard metabolic rate (SMR) and faster initial post-exercise recovery, but not with maximal metabolic rate (MMR) or aerobic scope (AS). (**A**) Mean SMR, MMR and AS (left to right) between the temperature-tolerant (red circles) and temperature-sensitive (blue squares) fish. (**B**) The temperature-tolerant group had significantly lower SMR compared with the temperature-sensitive fish (*t* = 2.69, *P* = 0.018). In addition, time to 63% recovery (τ, in hours) was lower in the temperature-tolerant compared with the temperature-sensitive group (*t* = 2.63, *P* = 0.020); *n* = 8 in each group. (**C**) This is illustrated by the typical example traces of post-exercise oxygen consumption rate from a temperature-tolerant (red, τ = 2.29 h) and a temperature-sensitive (blue, τ = 3.72 h) fish.

The temperature-tolerant fish displayed a significantly lower mean SMR compared with the temperature-sensitive group (*t* = 2.69, *P* = 0.018), with a mean SMR of 47.8 ± 1.1 mg O_2_ kg^−1^ h^−1^ in the temperature-tolerant group and 52.8 ± 1.6 mg O_2_ kg^−1^ h^−1^ in the temperature-sensitive group (Fig. 3A). However, no significant differences in MMR or AS were observed between the two groups (*t* = 1.48, *P* = 0.162 and *t* = 1.71, *P* = 0.110, respectively) with a mean MMR of 259.9 ± 15.1 mg O_2_ kg^−1^ h^−1^ in the temperature-tolerant group and 228.3 ± 15.1 mg O_2_ kg^−1^ h^−1^ in the temperature-sensitive group, and with a mean AS of 212.1 ± 15.1 mg O_2_ kg^−1^ h^−1^ in the temperature-tolerant group and 175.5 ± 15.3 mg O_2_ “kg^−1^ h^−1^ in the temperature-sensitive group (Fig. 3A). Interestingly, if AS is calculated as factorial scope (MMR/SMR), the temperature-tolerant group has a significantly higher scope (*t* = 2.37, *P* = 0.033) of 5.5 ± 0.3 compared with 4.4 ± 0.3 for the temperature-sensitive group. Fish from the temperature-tolerant group also had significantly faster initial post-exercise recovery (τ = 2.35 ± 0.19 h) compared with the temperature-sensitive individuals (τ = 3.13 ± 0.23 h; *t* = 2.63, *P* = 0.02; Fig. 3B). These parameters are clearly evident in the representative example EPOC curves (Fig. 3C). However, the total time to recovery varied greatly within both groups, but was not statistically different (*t* = 1.88, *P* = 0.081); with the temperature-tolerant fish requiring 15.17 ± 2.14 h to return to RMR, whereas the temperature-sensitive fish took on average 24.55 ± 4.50 h to recover. Additionally, the EPOC did not differ between the temperature-tolerant and the temperature-sensitive fish, with an average EPOC of 755.8 ± 39.7 and 847.0 ± 56.8 mg O_2_ kg^−1^, respectively (*t* = 1.32, *P* = 0.210).

### Relative ventricular mass and cardiac contractility

**Figure 4:**
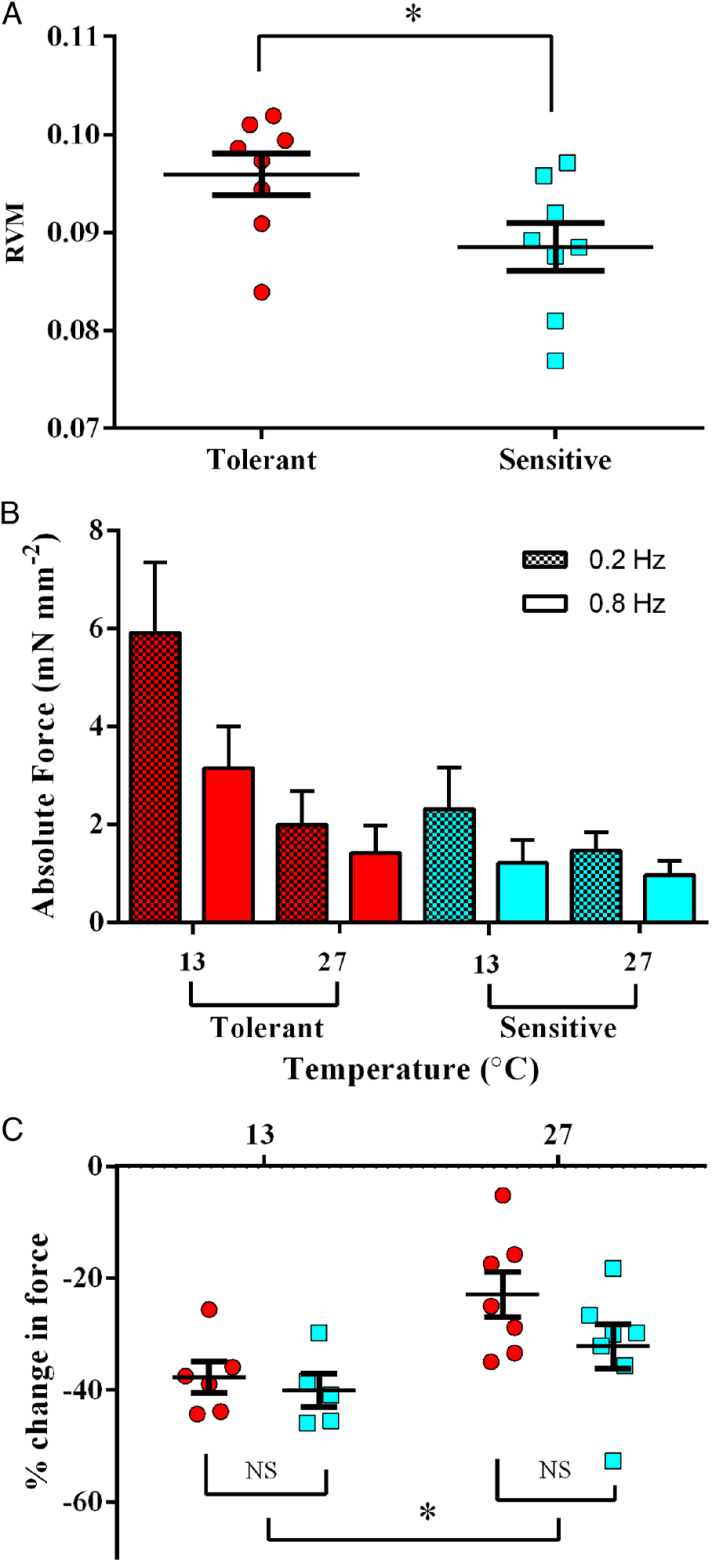
Temperature tolerance is related to relative ventricular mass (RVM) but not cardiac twitch force. (**A**) Temperature-tolerant fish had relatively bigger ventricles than the temperature-sensitive counterparts (*t* = 2.29, *P* = 0.038). (**B**) Absolute twitch force of the myocardial muscle preparations from temperature-tolerant fish (red bars) were higher, but not statistically so, at both experimental temperatures (12 and 27°C) and both stimulation frequencies (0.2 Hz, hatched bars; 0.8 Hz, open bars) when compared with preparations from temperature-sensitive hearts (blue bars). (**C**) The change in force of contraction at 12 and 27°C calculated as the percentage difference between force at 0.2 and 0.8 Hz. There was a significantly lower change in force of contraction in preparations stimulated at 27°C when compared with 12°C (*P* = 0.006), but there was no difference in twitch force between the two temperature tolerance groups (*P* = 0.131).

Temperature-tolerant fish had significantly larger hearts, with a mean RVM of 0.096 ± 0.002% compared with 0.089 ± 0.002% in the temperature-sensitive group (*t* = 2.29, *P* = 0.038; Fig. 4A). Myocardial preparations from temperature-tolerant fish tended to develop higher twitch force at 0.2 and 0.8 Hz compared with the preparations from temperature-sensitive fish at both 27 and 12°C; however, owing to the variability within the data, this difference was not statistically resolvable (Fig. 4B). To provide insight into the force–frequency relationship of the heart, the data were replotted as the relative change in force of contraction with change in stimulation frequency (calculated as the percentage change in force between 0.2 and 0.8 Hz; Fig. 4C). There was a significant difference in the effect of stimulation frequency on force development between the two experimental temperatures (*P* = 0.006), but no difference between the temperature-tolerant and temperature-sensitive groups (*P* = 0.131).

## Discussion

Using the European sea bass as a model species, our aim in this study was to explore the hypothesis that individual variation in thermal tolerance is associated with other key performance traits also involved in determining individual Darwinian fitness. We particularly examined aerobic metabolic performance (SMR, AMR, AS and EPOC) and cardiac contractility. Our data revealed that temperature-tolerant individuals displayed lower resting oxygen consumption rates (SMR) and faster initial recovery (i.e. lower τ) than the temperature-sensitive individuals. However, temperature tolerance was negatively corrected with maximal swimming speed (*U*_CAT_). There was no relationship between the temperature sensitivity of ventricular contractility and individual whole-organism temperature tolerance. Interestingly, we found that temperature-tolerant fish had larger hearts relative to body mass than temperature-sensitive fish.

### Temperature tolerance

The range of IULT for sea bass in this study (∼30–33°C) is similar to those reported for subtropical and tropical species (Schulte, 2007; Nilsson *et al.*, 2009) and those previously reported in sea bass (Claireaux *et al.*, 2013), but higher than those reported for other athletic, temperate species, such as salmonids (for review, see Farrell *et al.*, 2009). There is a well-documented relationship between thermal tolerance and body size in fish, with larger animals generally having lower thermal tolerance (for review, see Clarke and Johnston, 1999), but this is apparent only over a broad range of sizes, and our sea bass size differences were minimal. Considering the absence of differences between the two groups, there should be little to no influence of body mass on thermal tolerance within our experimental population.

### Swimming performance

We found a negative relationship between *U*_CAT_ and temperature tolerance (see Fig. 2B). This suggests a potential trade-off between temperature tolerance and swimming performance. However, within our population of sea bass, *U*_CAT_ showed no relationship with RVM, AS and τ (see online supplementary material Fig. 1C–F). This may indicate that these traits are not under high selective pressure. Also, the lack of a strong correlation between swimming performance and AS suggests that at these temperatures, a higher AS does not predict a better swimming performance. It is also important to keep in mind that a significant portion of *U*_CAT_ is attained anaerobically, and any trade-offs and/or compensatory processes between aerobic and anaerobic swimming are largely unknown. Yet, these may contribute to blur the relationship between maximal swimming speed and AS. It is, however, equally possible that *U*_CAT_ and temperature tolerance are not causally linked, but have causal links with a separate common factor. We had hypothesized this to be the cardiovascular system; however, our measures of ventricular muscle contractility do not support this conjecture (see the cardiovascular system and oxygen delivery subsection below). The *U*_CAT_ values in our study are similar to those previously reported for sea bass (Chatelier *et al.*, 2005; Nelson and Claireaux, 2005), Atlantic cod (*Gadus morhua*; Dutil *et al.*, 2007) and other fish of similar size and athleticism, such as salmonids (e.g. Webb, 1971; Day and Butler, 2005).

### Oxygen consumption

The values we report for SMR, MMR and AS compare well with previous studies on fish of similar size at acclimation temperatures similar to ours (Claireaux and Lagardère, 1999; Pörtner and Knust, 2007; Farrell *et al.*, 2009; Eliason *et al.*, 2011). Conversely, the EPOC and time to recovery were higher than those previously shown in fish (Scarabello *et al.*, 1991; Wood, 1991; Lee *et al.*, 2003). However, few studies (Reidy *et al.*, 2000; Hanson *et al.*, 2008; Roze *et al.*, 2013) have investigated the relationship between individual variation in temperature tolerance and individual variation in oxygen consumption within a (albeit small) population of fish. We found that temperature-tolerant individuals have a lower SMR, indicating more efficient metabolic performance than the temperature-sensitive group (i.e. they are more efficient metabolically at basic physiological functions; Killen *et al.*, 2014). The lower τ values (i.e. quicker initial recovery from exercise) in temperature-tolerant fish may also indicate better aerobic capacity and quicker recovery from oxygen debt. However, we did not find an association between individual temperature tolerance and individual MMR, AS, EPOC or total time to recovery. This suggests that oxygen supply and aerobic capacity at the acclimation temperature cannot be used as a predictor for temperature tolerance in this species.

### Cardiovascular system and oxygen delivery

A growing number of studies implicate the oxygen supply and delivery abilities of the fish cardiovascular system as the key determinant of fish distribution and population resilience in a changing thermal environment (Pörtner and Farrell, 2008; Farrell *et al.*, 2009; Eliason *et al.*, 2011). However, we found no correlation between the temperature sensitivity of ventricular contractility and individual whole-organism temperature tolerance. We did find that the temperature-tolerant individuals had significantly larger RVM than the temperature-sensitive individuals. This is in line with the findings of Anttila *et al.* (2013), who showed a positive association between large RVM and thermal tolerance in Atlantic salmon. Large RVM is associated with increased stroke volume (Farrell, 1991; Kluthe and Hillman, 2013), so it is possible that the temperature-tolerant fish had better blood supply, and therefore oxygen delivery, permitting extended cognitive function at higher temperatures. In support of this idea, we show a trend for increased ventricular muscle strip contractility in temperature-tolerant individuals. It is worth noting, however, that a large heart is metabolically costly and could be associated with a higher SMR. However, our temperature-tolerant fish had lower SMR, suggesting lower whole-body oxygen demand. Assessing tissue-specific oxygen consumption and *in vivo* cardiac function, including heart rate and cardiac output, would help to elucidate these relationships.

Bigger hearts should allow for improved blood and oxygen delivery to skeletal muscle for improved locomotor performance if they provide improved cardiac output. Chatelier *et al.* (2005) reported that sea bass swimming at *U*_CAT_ increased cardiac output almost exclusively through increased stroke volume, whereas Sandblom *et al.* (2005) found that sea bass swimming at 2 BL s^−1^ increased cardiac output exclusively through increased heart rate. However, bigger hearts also require more oxygen, and during extreme hypoxia or in an exhaustive exercise protocol, myocardial oxygenation is likely to be limited by venous blood oxygen content. Therefore, in the sea bass, where the heart is entirely composed of spongy tissue, our data showing poorer swimming ability in fish with slightly bigger hearts may not be surprising.

### Conclusions and perspectives

The European sea bass is one of the most sought-after fish for both commercial and recreational fishermen in Europe. This has led to unsustainable fishing practices, and the sea bass stocks have been declining, so much so that the European Commission has implemented new management strategies in order to avert a complete stock collapse. In addition, any potential effects of environmental stressors associated with climate change may exacerbate the threats imposed on the sea bass populations. Understanding the physiological basis and limitations for tolerance to these stressors, such as temperature, is therefore of the upmost importance.

Our study found that temperature-tolerant sea bass had larger but not more powerful hearts, lower SMR and faster initial recovery after exercise. They also had poorer *U*_CAT_, hinting at a potential trade-off between these two performance indices. Our finding that whole-animal temperature tolerance is correlated with cardiac size could suggest that selection on the cardiorespiratory system may benefit thermal tolerance (see Anttila *et al*., 2013; McBryan *et al*., 2013). However, it is worth remembering the complex natural environment in which a fish lives; temperature and water velocity, along with other biotic and abiotic factors, exert controlling, limiting, masking, lethal and directive effects on a fish's physiology, and causality between factors is difficult to delineate.

## Supplementary material

Supplementary material is available at *Conservation Physiology* online.

## Funding

This work was supported by European Cooperation in Science and Technology (COST) action COST FA1004 ‘Conservation Physiology of Marine Fishes’ short term scientific missions grant (reference number: COST-STSM-FA1004-11440).

## Supplementary Material

Supplementary Data
