# Supplementary material for: Intraspecific individual variation of temperature tolerance associated with oxygen demand in the European sea bass (Dicentrarchus labrax)
Source: Conserv Physiol. 2016 Jan 8;4(1):cov060. doi: 10.1093/conphys/cov060 (PMC4922261; doi:10.1093/conphys/cov060)
Supplement: Supplementary Data [file cov060supp.zip › cov060supp.docx]

**S1**. No significant relationship between swimming capacity and fish mass (A), body length (B), standard metabolic rate (C), aerobic scope (D), τ (E), or relative ventricular mass (F). Each data point is an individual fish, red circles are the temperature-tolerant individuals, blue squares are the temperature-sensitive fish (n=16). Pearson’s correlation statistical parameters (R^2^ and *p*-value) are shown for each panel.
